# GluN1 hypomorph mice exhibit wide-ranging behavioral alterations

**DOI:** 10.1111/j.1601-183X.2012.00767.x

**Published:** 2012-04

**Authors:** C Barkus, L A Dawson, T Sharp, D M Bannerman

**Affiliations:** † Department of Experimental PsychologyOxford OX1 3UD, UK; ‡ Department of Pharmacology, University of OxfordOxford OX1 3UD, UK; § Eisai Ltd, Neurosciences Product Creation UnitHatfield, UK

**Keywords:** Anxiety, cognition, GluN1, mice, schizophrenia

## Abstract

The psychotomimetic effects of *N*-methyl-d-aspartate receptor (NMDA) antagonists such as ketamine and phencyclidine suggest a role for reduced NMDA receptor-mediated neurotransmission in schizophrenia. GluN1 ‘hypomorph’ (GluN1^hypo^) mice exhibit reduced NMDA receptor expression and have been suggested as a mouse model of schizophrenia. However, NMDA receptors are ubiquitous and are implicated in many physiological and pathological processes. The GluN1^hypo^ mice have a global reduction of NMDA receptors and the consequences of such a global manipulation are likely to be wide-ranging. We therefore assessed GluN1^hypo^ mice on a battery of behavioral tests, including tests of naturalistic behaviors, anxiety and cognition. GluN1^hypo^ mice exhibited impairments on all tests of cognition that we employed, as well as reduced engagement in naturalistic behaviors, including nesting and burrowing. Behavioral deficits were present in both spatial and non-spatial domains, and included deficits on both short- and long-term memory tasks. Results from anxiety tests did not give a clear overall picture. This may be the result of confounds such as the profound hyperactivity seen in GluN1^hypo^ mice, although hyperactivity cannot account for all of the results obtained. When viewed against this background of far-reaching behavioral abnormalities, the specificity of any one behavioral deficit is inevitably called into question. Indeed, the present data from GluN1^hypo^ mice are indicative of a global impairment rather than any specific disease. The deficits seen go beyond what one would expect from a mouse model of schizophrenia, thus questioning their utility as a selective model of this disease.

Schizophrenia is a psychiatric condition characterized by positive symptoms such as hallucinations and delusions, negative symptoms such as flattened affect, and cognitive deficits (DSM IV-TR; American Psychiatric Association 2000). Dopamine is thought to have a prominent role in the pathology of this disease, but there is also substantial evidence for a role for glutamate in schizophrenia (Carlsson *et al.* 2004; Javitt 2007). For instance, altered levels of glutamate have been found in the frontal cortex and hippocampus of schizophrenic patients (Tsai *et al.* 1995) and the psychotomimetic effects of drugs such as ketamine and phencyclidine form part of this body of evidence (Javitt & Zukin 1991). Both these agents act as antagonists to the *N*-methyl-d-aspartate receptor (NMDA) class of glutamate receptor and these ionotropic receptors have a central role in the glutamate hypothesis of schizophrenia.

While these acute pharmacological interventions which reduce NMDA receptor functionality provide important information, schizophrenia is thought to involve neurodevelopmental alterations (Harrison 1997; Raedler *et al.* 1998). Such life-long manipulations can be investigated through the use of mutant mouse lines. However, investigations into the behavioral consequences of reduced NMDA receptor function from conception and throughout the lifetime of the animal have been hampered by the perinatal lethality of the complete NMDA knockout (KO) manipulation (Forrest *et al.* 1994). However, mice expressing 5–10% levels of the obligatory GluN1 NMDA receptor subunit, and consequently having 5–10% normal levels of functional NMDA receptors, have been produced and are viable (Mohn *et al.* 1999). These GluN1 hypomorph (GluN1^hypo^) mice have been suggested as a mouse model of schizophrenia and have previously been shown to exhibit behavioral phenotypes relevant to schizophrenia such as deficits in prepulse inhibition (PPI, Duncan *et al.* 2004, 2006a,b; Fradley *et al.* 2005; Moy *et al.* 2006), reduced social interaction (Duncan *et al.* 2004; Halene *et al.* 2009; Mohn *et al.* 1999), hyperactivity (Duncan *et al.* 2006b; Mohn *et al.* 1999) and increased stereotypy (Mohn *et al.* 1999). Furthermore, these alterations can be influenced by antipsychotic treatment (Duncan *et al.* 2006a,b; Mohn *et al.* 1999). Taken together, these results suggest that these mice display many behavioral alterations considered analogous to several aspects of schizophrenia.

However, these deficits can only be fully interpreted when viewed against a more complete description of the behavioral phenotype of GluN1^hypo^ mice. Only then can the selectivity of the model, and hence its utility, be judged. The GluN1^hypo^ mice have a global reduction of NMDA receptors. NMDA receptors have been implicated in a great many processes in a variety of different brain areas. The consequences of such a global manipulation are therefore likely to be wide-ranging. Here, we report the results from tests of naturalistic behaviors, cognition and anxiety in GluN1^hypo^ mice and their wild-type (WT) littermates. This study was carried out to extend the behavioral phenotyping of these mice in order to assess the suitability of GluN1^hypo^ mice as a model of schizophrenia.

## Materials and methods

### Animals

Male and female GluN1 hypomorph mice (GluN1^hypo^, Mohn *et al.* 1999) and their WT littermates, maintained on a 129S9/SvEvH x C57Bl/6J F1 background were obtained from GlaxoSmithKline (LAS Department, Hertfordshire, UK). The mice used were a product of heterozygote × heterozygote crosses. Before testing, the mice were housed at the University of Oxford in holding rooms on a 12/12-h light/dark cycle (lights on at 0700 h and off at 1900 h). Mice were housed in groups of 3 with *ad libitum* food and water (unless otherwise stated), and were typically 3 months of age at the start of testing. All testing was performed during the light phase. The maintenance and testing of these animals were performed under the auspices of the UK Home Office laws and guidelines for the treatment of animals under scientific procedures and the local ethical review board at the University of Oxford.

### Behavioral protocols

A first experimentally naÏve cohort of 29 WT (14 males and 15 females) and 30 GluN1^hypo^ mice (15 males and 15 females) were tested in the following order: elevated plus maze, light/dark box, open field, hyponeophagia, discrete trial spontaneous alternation in a T-maze, spatial novelty preference in a Y-maze, nesting, food and water consumption, burrowing, locomotor activity, sucrose preference, social interaction following habituation to the test environment and associative long-term spatial reference memory on an elevated Y-maze (appetitively motivated). A separate cohort of experimentally naÏve animals were tested on social interaction (with no habituation to the test environment; 28 WT mice, 14 males and 14 females; 26 GluN1^hypo^ mice, 14 males and 12 females), object recognition (29 WT mice, 15 males and 14 females; 26 GluN1^hypo^ mice, 14 males and 12 females) followed by an associative, long-term memory visual discrimination task (appetitively motivated; 29 WT mice, 14 males and 15 females; 21 GluN1^hypo^ mice, 10 males and 11 females). At least a 2-day gap was left between each behavioral test and all tests were assessed with the experimenter blind to genotype. Testing was typically performed between 0900 h and 1500 h unless otherwise stated.

GluN1^hypo^ mice were noted to persistently scratch, usually in the neck/flank region, which has also been seen by other laboratories (Moy *et al.* 2008). In some cases, this behavior led to the appearance of an open wound at which point the animal was culled. This accounts for the slow decline in numbers across the behavioral experiments.

### Tests of naturalistic behavior

#### Nesting

An assessment of nesting was performed as described previously (Deacon 2006a). Briefly, mice (14 WT males, 15 WT females, 12 GluN1^hypo^ males and 15 GluN1^hypo^ females) were individually housed 1 h before the onset of the dark phase with no enrichment aside from a 3-g square of pressed cotton (a ‘nestlet’ that makes up part of normal cage enrichment). Nests were assessed the following morning at 0900 h using the following rating scale:

1. Nestlet not noticeably touched (>90% intact).
2. Nestlet partially torn up (50–90% remaining intact).
3. Nestlet mostly shredded but no identifiable nest site.
4. An identifiable, but flat nest.
5. A perfect (or near-perfect) nest.

#### Food and water consumption

Food and water consumption were measured by placing mice (14 WT males, 15 WT females, 12 GluN1^hypo^ males and 15 GluN1^hypo^ females) into individual, clean cages complete with enrichment, 6 g of food pellets and a water bottle with a measured amount of water 1 h before the onset of the dark cycle. The food and water remaining the next morning were then assessed. This was repeated twice on consecutive days and the values averaged.

#### Burrowing

Burrowing behavior was assessed similar to the method described previously (Deacon 2006b). A cylinder of dark gray plastic (6.8 cm diameter, 20 cm in length), with the open end raised by 3 cm was filled with 200-g food pellets [Special Diet Services (Braintree, UK) RM1-pelleted diet, product number 801151, which is also used as normal food chow for these mice], and placed in a clean cage with a thin layer of bedding. Mice (14 WT males, 15 WT females, 12 GluN1^hypo^ males and 15 GluN1^hypo^ females) were placed in the cage 3 h before the onset of the dark cycle. The amounts of pellets remaining in the tube were measured after 2 h. The tubes were refilled following the 2-h measurement and placed back into the cage. The mice were then left until 0900 h the following morning when the amount of pellets remaining was again recorded to give the overnight measure. This refilling of the tubes after the 2-h timepoint, which differs from the previously published protocol, was carried out because of the generally very high levels of burrowing noted after this shorter period of time.

#### Locomotor activity

An automated beam-break system was used to assess locomotor activity over a 2-h period. Mice (14 WT males, 15 WT females, 12 males GluN1^hypo^ and 15 GluN1^hypo^ females) were placed in transparent plastic cages (26 × 16 × 17 cm), fitted with ventilated lids. Two infra-red beams crossed the width of the cage floor, each 7 cm from the center of the cage, 1.5 cm above the floor. The total number of ‘crossovers’ (breaking the two beams consecutively) during this time was used as a measure of larger locomotor movements that excluded small, stereotyped movements.

#### Sucrose preference

Sucrose preference was performed as previously described (Barkus *et al.* 2011). Briefly, mice (14 WT males, 15 WT females, 11 males GluN1^hypo^ and 14 GluN1^hypo^ females) were individually housed in large cages with enrichment for 8 days with *ad libitum* access to food and two bottles. During the first 4 days, both bottles contained water. During the subsequent 4 days, one bottle contained water and the other contained an 8% (weight/volume) sucrose solution. The relative positions of the bottles were counter-balanced across mice and switched after every 2 days to prevent development of a place preference. Average water and sucrose consumption across the 4-day period was calculated and sucrose preference derived from these by dividing sucrose consumption by total fluid consumption [sucrose/(sucrose + water)].

### Tests of anxiety

#### Elevated plus maze

Mice (14 WT males, 15 WT females, 15 GluN1^hypo^ males and 15 GluN1^hypo^ females) were placed in the center and allowed to explore a plus-shaped maze for 5 min. The maze had arms of 35 × 6 cm leading from a 6 × 6 cm center square. There were two closed arms (10 lux) with 20-cm high walls and two open arms (85 lux) with 0.5-cm high walls (Handley & Mithani 1984; Lister 1987; Pellow *et al.* 1985). The maze was elevated by 70 cm from the ground and Ethovision XT (Noldus, Wageningen, The Netherlands) was used to record time spent in, latency to enter and number of entries into, the open arms as measures of anxious behavior. Total distance covered was also measured by Ethovision.

#### Light/dark box

The light/dark box (Crawley 1981; Crawley & Goodwin 1980) consisted of an open white compartment (30 × 20 × 20 cm, 900 lux) joined by a 3 × 3 cm opening to a dark compartment (15 × 20 × 20 cm, 0 lux) which was painted black and covered with a lid. The anxiogenic nature of the white compartment was increased by additional illumination from a 60-W angle poise lamp placed 45 cm above the center of the apparatus. The mice used were either started in the light section (six WT males, six WT females, six GluN1^hypo^ males and six GluN1^hypo^ females) or the dark section (eight WT males, nine WT females, nine GluN1^hypo^ males and nine GluN1^hypo^ females). The mice were placed in the middle of the section facing away from the opening and behavior scored for 5 min. The latency to cross (defined by all four feet entering the compartment), time spent in each section (again defined by all four feet being in that area), and the number of transitions through the opening were measured.

#### Open field

Behavior was assessed in a white, anxiogenic open field (60 cm diameter, 2800 lux). Ethovision XT (Noldus) was used to split the arena into a central area of 10 cm radius and an outer area of 20 cm radius (Hall 1936; Treit & Fundytus 1988). Mice (14 WT males, 15 WT females, 14 GluN1^hypo^ males and 15 GluN1^hypo^ females) were placed into the apparatus at the edge and the distribution of time spent in the central and peripheral areas was taken as a measure of anxiety. Total distance traveled was also recorded.

#### Hyponeophagia

The hyponeophagia test of anxiety relies on the approach/avoidance conflict that occurs when an animal is presented with a novel foodstuff in a novel (and potentially dangerous) environment (Burns *et al.* 1996). The apparatus consisted of an upturned translucent plastic jug (15 cm in diameter) with a spout protruding a further 2 cm in which a food well was located. The food well contained condensed milk diluted 50/50 with water. Mice (14 WT males, 15 WT females, 14 GluN1^hypo^ males and 15 GluN1^hypo^ females), naÏve to milk, were food restricted overnight to 1 g/mouse and tested the following morning. When tested, they were placed facing away from the well and the jug gently lowered into position (in 110-lux light levels). The latency to drink was taken with a cut-off time of 2 min. Mice that did not drink in this time were individually housed for approximately 3 min before being retested. Mice were tested a maximum of three times, the cumulative latency to begin to drink the milk being used where necessary. Mice were not retested once they had consumed the novel foodstuff. Following testing, mice were held in a separate cage from their homecage to prevent the social transmission of food preference.

#### Social interaction

Two mice of the same sex and genotype (from a pool of 14 WT male, 14 WT female, 14 GluN1^hypo^ male and 12 GluN1^hypo^ female mice) that had previously been housed in separate cages, and therefore were naÏve to one another, were placed in a clean cage (41.5 × 25.5 × 11.5 cm) for 10 min in low light levels (20 lux). Bedding sufficient to cover the bottom of the cage was present and changed for each trial. This protocol was repeated on two consecutive days, with novel pairings being used on each day. These encounters were recorded and scored blind with respect to genotype to give a measure of social interaction in a novel environment. The social behaviors included were sniffing, grooming and following, while aggressive behavior was defined as biting, scratching and chasing.

Differences in the levels of social investigation between groups could be due to different amounts of exploration of the test environment, which might be expected to compete with social behaviors. For example, we have previously shown that the apparent deficit in social behavior in the GluA1 KO mouse can be rescued with extensive habituation to the testing environment (Barkus *et al.* 2011). To investigate this possibility, a separate cohort of mice (12 WT male, 14 WT female, 10 GluN1^hypo^ male and 14 GluN1^hypo^ female mice) were extensively habituated to the testing arena before assessment of social behavior, by individual exposure to the testing apparatus for 10 min/day for a period of 5 days. Litter was once again present and changed for every trial. Social interaction testing was then conducted on the sixth and seventh days using the protocol described above.

### Tests of cognition

Learning and memory were assessed using a variety of tests, relying on either spontaneous, exploratory behaviors or requiring appetitive motivation. Although the battery of learning tests used is by no means exhaustive, our aim was to assess both short-term and long-term memory, in both spatial and non-spatial domains.

#### Discrete trial, spontaneous alternation (enclosed T-maze)

A T-shaped maze made of wood painted dark gray with 30 × 10 × 29 cm arms, with a central partition extending 7 cm into the start arm from the back of the maze, was used to assess spontaneous alternation (Deacon & Rawlins 2006; Dember & Fowler 1958; Lalonde 2002). Mice were placed into the maze facing the wall of the start arm and allowed to make a free choice of either goal arm. The mouse was then restricted to that goal arm for 30 s by use of a guillotine door. The central partition was then removed and all doors reopened. The mouse was again placed at the end of start arm facing the wall and allowed to make a further free choice of either goal arm. This was performed twice a day for five consecutive days with an approximate interval of 4 h between the two daily sessions. Whether or not the animal chose the novel arm on the second run (i.e. whether it alternated) was recorded and summed across 10 trials.

#### Spatial novelty preference in a Y-maze

Spatial novelty preference was assessed in an enclosed Perspex Y-maze as described previously (Sanderson *et al.* 2007). Briefly, a Perspex Y-maze with arms of 30 × 8 × 20 cm was placed into a room containing a variety of extramaze cues. Mice (14 WT males, 15 WT females, 12 GluN1^hypo^ males and 15 GluN1^hypo^ females) were assigned two arms (the ‘start’ and the ‘other’ arm) to which they were exposed during the first phase (the exposure phase), for 5 min. This selection of arms was counterbalanced with respect to genotype. Timing of the 5-min period began only once the mouse had left the start arm. The mouse was then removed from the maze and returned to its homecage for a 1-min interval between the exposure and test phases. During the test phase, mice were allowed free access to all three arms. Mice were placed at the end of the start arm and allowed to explore all three arms for 2 min beginning once they had left the start arm. An entry into an arm was defined by a mouse placing all four paws inside an arm. Similarly, a mouse was considered to have left an arm if all four paws were placed outside the arm. The times that mice spent in each arm were recorded manually and a novelty preference ratio was calculated for the time spent in arms [novel arm/(novel + other arm)].

#### Spatial reference memory on the elevated Y-maze

A Y-shaped maze consisting of a central polygonal area (14 cm in diameter), and three arms (each 50 × 9 cm with 0.5-cm high walls), elevated 80 cm from the ground, was used in this spatial reference memory task. A small food well located 5 cm from the distal end of each arm contained the 50% condensed milk reward which was available if the mouse located the correct target arm.

The mice (14 WT males, 14 WT females, 9 GluN1^hypo^ males and 14 GluN1^hypo^ females) were initially habituated to drinking the milk reward from the maze in the colony holding room (i.e. not the experimental testing room), first in groups and then individually. Each mouse was then assigned a target arm in which the milk reward was always located, defined by allocentric spatial cues. Assignment of mice to target arms was counterbalanced with respect to genotype. Either one of the other two arms could be used as a start arm. Each mouse received 10 trials a day with 5 trials starting from each of the non-target arms in a pseudorandom sequence, with a maximum number of three consecutive identical arm starts. Mice were allowed to make a single arm entry and drink the reward if the correct choice was made, before being removed from the maze. Mice that chose incorrectly were removed immediately without reward. Each mouse received 10 trials/day on 10 consecutive days with an approximate intertrial interval (ITI) of 15 min and the number of correct choices per day was recorded. The maze was periodically rotated to prevent the use of intramaze cues but the position of the reward remained constant relative to allocentric spatial cues. Mice were maintained at a level of food deprivation sufficient to run readily on the maze and never below 85% of free-feeding weight. On the final day of testing (day 10), to ensure that the reward itself was not providing an olfactory cue, the rewarded arm was only baited after the arm choice had been made by the mice (post-choice baiting).

#### Object recognition

Object recognition was performed in a gray arena (40 × 40 × 40 cm) under low light levels (20 lux). Mice (15 WT males, 14 WT females, 14 GluN1^hypo^ males and 12 GluN1^hypo^ females) were habituated to the empty test arena for 10 min/day on the 3 days preceding the test. For the training phase, two identical objects (A_1_ and A_2_) were placed in the arena 10 cm away from two adjacent corners and mice allowed to explore freely for 10 min. The mice were placed facing the center of the wall opposite the objects. After an ITI of 1 h, mice were reintroduced to the testing arena for the test phase, T2, which lasted 5 min. During the test phase, the arena contained one duplicate object, identical to those used during training (A_3_), plus one novel object (B_1_). The identities and spatial positions of the novel and familiar objects were counterbalanced relative to genotype and sex. The arena and objects were wiped down with 70% ethanol between trials to minimize olfactory cues.

Object exploration was scored manually and defined as the animal being within 1 cm of the object and orientated toward it. Rearing against the object was counted as exploration but not sitting on the object (rarely observed). Example objects include tin cans and plastic water bottles. The ratio between the time spent exploring the novel object vs. total time spent exploring the objects [novel/(novel + familiar)] was taken as a measure of object memory. Total exploration time of the sample objects during the exposure phase was also calculated to determine whether the two groups had equal exposure to the familiar object.

#### Visual discrimination learning

Non-spatial, associative, long-term memory was assessed using a simple, appetitively motivated visual discrimination task. Mice were trained to associate a reward (50% condensed milk) with one of two visually cued/patterned goal arms (gray or black-and-white striped arms) in a T-maze. Mice were assigned to one of the patterned goal arms and were always rewarded only in that goal arm. Testing took place in an enclosed T-maze with arms of 10 × 30 × 30 cm. The right/left spatial position of these rewarded and non-rewarded arms was altered in a pseudorandom order to remove any spatial component to the task, with a maximum of three consecutive identical arm positions and an equal number of each position within a block of 10 trials. Mice were extensively habituated to the maze and to the milk reward before testing using a pair of neutral colored goal arms. They then received 10 trials/day for 8 days with an approximate ITI of 15 min and the total number of correct choices per day was recorded. On the final day of testing (day 8), to ensure that the reward itself was not providing an olfactory cue, the rewarded arm was only baited after the arm choice had been made by the animal.

### Statistical analysis

Results were analyzed using univariate anovas with sex and genotype taken as between subject factors except in the following cases. As nesting and spontaneous alternation generated non-continuous values, these were analyzed using Mann–Whitney *U* tests with the genotypes collapsed across sex. The social interaction test was performed in different groups of mice that had either been habituated to the test environment or were naÏve to it, and so habituation was taken as an additional between subject factor in the anova. Aggression in the social interaction tests was seen only in male mice, and so female mice and the factor of sex were excluded from this analysis. Performance in cognitive tasks was tested using either univariate anovas as above or repeated measures anovas with test session as the within subject factor as appropriate. Additionally, cognitive performance was tested against chance using one-sample *t*-tests with the genotypes collapsed across sex in spontaneous alternation, spatial novelty preference Y-maze, object recognition, spatial reference memory and the visual discrimination task. When interactions were significant, these were further explored either using post-hoc pairwise comparisons with Bonferroni corrections in the case of interactions between different between subject factors or by using simple main effects analysis when a within subject factor was involved in the interaction.

All statistical analysis was carried out using SPSS version 15 and *P* < 0.05 considered significant.

## Results

### Tests of naturalistic behavior

#### Nesting and burrowing

In the assessments of naturalistic behavior, GluN1^hypo^ mice displayed deficits in both nesting [nesting scores (median ± IQR): WT 5 (4.5–5), GluN1^hypo^ 3.5 (1.5–5); *U*_(56)_ = 190.500, *P* < 0.001] and burrowing behavior after 2 h [amount burrowed (mean ± SEM): WT 140.3 ± 14.0 g, GluN1^hypo^ 6.3 ± 3.5; *F*_1,52_ = 87.685, *P* < 0.001] and when left overnight (amount burrowed: WT 140.0 ± 6.3 g, GluN1^hypo^ 10.6 ± 5.7 g; *F*_1,52_ = 229.585, *P* < 0.001). Sex was also found to affect burrowing at the 2-h time point (*F*_1,52_ = 4.810, *P* = 0.033), with male mice (95.7 ± 17.6 g) burrowing more than female mice (58.4 ± 15.1 g). However, sex did not interact with genotype at either time point (2 h *F*_1,52_ = 1.511, *P* = 0.225; overnight *F*_1,52_ = 0.769, *P* = 0.385).

#### Food and water intake

The GluN1^hypo^ mice ate more daily than WT mice [pellet consumption (mean ± SEM): WT 1.9 ± 0.1 g; GluN1^hypo^ 2.6 ± 0.1 g; *F*_1,52_ = 22.943, *P* < 0.001] but water consumption was not altered [daily water intake (mean ± SEM): WT 3.6 ± 0.1 ml; GluN1^hypo^ 3.7 ± 0.4 ml; *F*_1,52_ = 0.006, *P* = 0.940]. No effect of sex (food intake *F*_1,52_ = 2.292, *P* = 0.136; water intake *F*_1,52_ = 0.932, *P* = 0.339) or genotype × sex interaction (food intake *F*_1,52_ = 0.006, *P* = 0.938; water intake *F*_1,52_ = 1.625, *P* = 0.208) was seen in either measure.

#### Locomotion

The GluN1^hypo^ mice displayed pronounced locomotor hyperactivity in photobeam activity cages, making many more beam crossovers than WT mice (crossover beam breaks in 2-h session (mean ± SEM): WT 106.4 ± 9.7; GluN1^hypo^ 764.0 ± 51.8; *F*_1,52_ = 164.115, *P* < 0.001). No effect of sex (*F*_1,52_ = 0.717, *P* = 0.401) or genotype × sex interaction (*F*_1,52_ = 0.283, *P* = 0.597) was seen.

#### Sucrose preference

As noted above, water intake did not differ between genotypes, although the measured water intake in this experiment was markedly increased from the single-bottle paradigm used previously [daily water intake (mean ± SEM): WT 10.9 ± 0.3 ml; GluN1^hypo^ 10.2 ± 0.3 ml; *F*_1,50_ = 2.355, *P* = 0.131]. Again, no effect of sex (*F*_1,50_ = 0.403, *P* = 0.528) or genotype × sex interaction (*F*_1,50_ = 0.128, *P* = 0.722) was seen on this measure. However, an increase in sucrose intake was seen in the GluN1^hypo^ mice as compared to the WT mice [daily sucrose solution intake (mean ± SEM): WT 25.7 ± 0.9 ml; GluN1^hypo^ 29.7 ± 1.5 ml; *F*_1,50_ = 6.680, *P* = 0.013], which drives an increase in the sucrose preference ratio [preference ratio (mean ± SEM): WT 0.68 ± 0.01; GluN1^hypo^ 0.72 ± 0.01; *F*_1,50_ = 6.386, *P* = 0.015].

No effect of sex was seen in either of these measures (sucrose solution intake *F*_1,50_ = 0.168, *P* = 0.684; preference ratio *F*_1,50_ = 0.046, *P* = 0.832). A genotype × sex interaction was seen on sucrose intake (*F*_1,50_ = 4.842, *P* = 0.032). Pairwise comparisons showed WT male mice consumed less sucrose solution than GluN1^hypo^ male mice (WT 24.1 ± 1.0 ml; GluN1^hypo^ 32.1 ± 2.6 ml; *P* = 0.012) but no difference was seen between female mice (WT 27.1 ± 1.5 ml; GluN1^hypo^ 27.7 ± 1.7 ml; *P* = 1.000). No other differences were seen (WT male vs. WT female mice *P* = 1.000; GluN1^hypo^ male mice vs. GluN1^hypo^ female mice *P* = 0.492; WT male vs. GluN1^hypo^ female mice *P* = 0.726; WT female vs. GluN1^hypo^ male mice *P* = 0.264). This was not sufficient to produce a genotype × sex interaction on the preference ratio (*F*_1,50_ = 1.589, *P* = 0.213).

### Tests of anxiety

#### Elevated plus maze

GluN1^hypo^ mice showed no changes in the time spent in the open arms of the elevated plus maze (EPM) [time in open arms (mean ± SEM): WT 8.3 ± 2.30 s, GluN1^hypo^ 12.5 ± 5.4 s; effect of genotype *F*_1,55_ = 0.459, *P* = 0.501; effect of sex *F*_1,55_ = 0.003, *P* = 0.959; genotype × sex interaction *F*_1,55_ = 0.085, *P* = 0.771]. Latency to first enter the open arm (mean ± SEM: WT 143.2 ± 21.23 s, GluN1^hypo^ 139.84 ± 24.83 s; *F*_1,55_ = 0.005, *P* = 0.946), and number of entries into the open arms (mean ± SEM: WT 5.1 ± 0.99, GluN1^hypo^ 7.7 ± 2.42; *F*_1,55_ = 0.923, *P* = 0.341) were also not affected by genotype although an effect of sex was seen in latency (*F*_1,55_ = 4.215, *P* = 0.045) with female mice (179.9 ± 23.4 s) taking longer to make their first entry to the open arm than male mice (108.0 ± 21.2 s). No genotype × sex interaction was seen (*F*_1,55_ = 0.021, *P* = 0.886) and no main effect of sex (*F*_1,55_ = 1.190, *P* = 0.280) or interaction was seen in number of open arm entries (*F*_1,55_ = 0.081, *P* = 0.777). Genotype did, however, affect the total distance moved during the test, with GluN1^hypo^ mice being hyperactive (mean ± EM: WT 1373.3 ± 48.41 cm, GluN1^hypo^ 1815.5 ± 78.83 cm; *F*_1,55_ = 21.704, *P* < 0.001) with no effect of sex (*F*_1,55_ = 0.024, *P* = 0.877) or genotype × sex interaction (*F*_1,55_ = 0.002, *P* = 0.967).

#### Light/dark box

In the light/dark box, GluN1^hypo^ mice spent more time in the light area than WT mice [time spent in light area (mean ± SEM): WT 32.6 ± 8.8 s GluN1^hypo^ 114.4 ± 33.26 s; *F*_1,20_ = 5.371, *P* = 0.031] when started in the light and this was unaffected by sex (effect of sex *F*_1,20_ = 0.125, *P* = 0.727; genotype × sex interaction *F*_1,20_ = 0.759, *P* = 0.394). GluN1^hypo^ mice also took longer to move into the dark area when started in the light [latency to dark area (mean ± SEM): WT 18.3 ± 6.8 s GluN1^hypo^ 111.3 ± 33.9 s; *F*_1,20_ = 6.900, *P* = 0.016], which was also unaffected by sex (effect of sex *F*_1,20_ = 0.326, *P* = 0.575; genotype × sex interaction *F*_1,20_ = 0.684, *P* = 0.418). The number of transitions made did not differ between groups (WT 1.8 ± 0.7 GluN1^hypo^ 2.9 ± 0.8; effect of genotype *F*_1,20_ = 1.266, *P* = 0.274; effect of sex *F*_1,20_ = 2.093, *P* = 0.163; genotype × sex interaction *F*_1,20_ = 0.103, *P* = 0.751).

However, genotype effects on time spent in the light area were not seen when started in the dark (WT 4.6 ± 1.8 s, GluN1^hypo^ 34.0 ± 15.4 s; *F*_1,31_ = 3.629, *P* = 0.066) although there was a strong trend for the GluN1^hypo^ mice to once again spend more time in the light area. No genotype effect was seen on latency to enter the light area when started in the dark section (WT 220.7 ± 26.5 s, GluN1^hypo^ 175.0 ± 28.1 s; *F*_1,31_ = 1.403, *P* = 0.245). Effects of sex were also absent (time spent in light area; effect of sex *F*_1,31_ = 1.614, *P* = 0.213; genotype × sex interaction *F*_1,31_ = 0.806, *P* = 0.376; latency to light area; effect of sex *F*_1,31_ = 0.010, *P* = 0.921; genotype × sex interaction *F*_1,31_ = 0.405, *P* = 0.529). When started in the dark section, GluN1^hypo^ mice were seen to make more transitions than WT mice (WT 0.8 ± 0.3, GluN1^hypo^ 2.8 ± 0.8; *F*_1,31_ = 5.071, *P* = 0.032) but this was not affected by sex (effect of sex *F*_1,31_ = 0.232, *P* = 0.634; genotype × sex interaction *F*_1,31_ = 0.405, *P* = 0.529).

#### Open field

In the open field, GluN1^hypo^ mice spent more time in the center area than WT mice [time in center (mean ± SEM): WT 3.4 ± 0.4 s, GluN1^hypo^ 5.9 ± 1.0 s; *F*_1,54_ = 7.159, *P* = 0.010], implying that they are less anxious in this test. An effect of sex was seen on the time spent in the center (*F*_1,54_ = 9.225, *P* = 0.004) with male mice (6.2 ± 0.9 s) spending more time in the center than female mice (3.2 ± 0.6 s), but this did not interact with genotype (*F*_1,54_ = 2.665, *P* = 0.108). GluN1^hypo^ mice were also observed to cover a greater distance during the test than WT mice [distance moved (mean ± SEM): WT 2442.2 ± 68.69 cm, GluN1^hypo^ 3279.4 ± 167.7 cm; *F*_1,54_ = 20.585, *P* < 0.001). When this is taken as a covariate, the difference between the genotypes in time spent in the center no longer remains (*F*_1,53_ = 1.728, *P* = 0.194). No effect of sex (*F*_1,54_ = 0.153, *P* = 0.697) or genotype × sex interaction (*F*_1,54_ = 0.521, *P* = 0.473) was seen on distance moved.

#### Hyponeophagia

GluN1^hypo^ mice took longer before drinking in the hyponeophagia test (latency to drink (mean ± SEM): WT 81.2 ± 18.5 s, GluN1^hypo^ 266 ± 22.1 s; *F*_1,54_ = 40.152, *P* < 0.001), which could be taken to imply that they are more anxious in this test. No effect of sex (*F*_1,54_ = 0.654, *P* = 0.422) or genotype × sex interaction (*F*_1,54_ = 0.006, *P* = 0.941) was seen on this measure.

#### Social interaction

**Figure 1:**
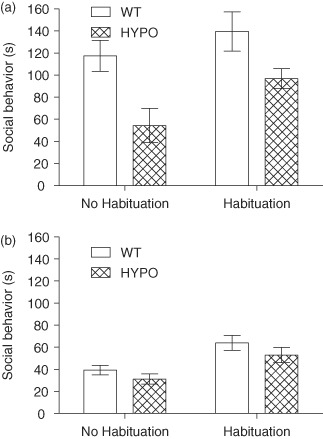
Time spent socially interacting for GluN1 ^hypo^(HYPO) and WT mice split into male (a) and female (b) mice. In the social interaction task, GluN1^hypo^ mice show a deficit in social behavior that is driven by differences between male mice (a) and not female mice (b). This group difference was not diminished by habituation to the testing environment but did increase social activity in all mice. Time spent socially interacting is expressed as mean ± SEM.

A main effect of genotype was seen for social interaction (*F*_1,96_ = 15.748, *P* < 0.001) with WT mice interacting more than GluN1^hypo^ mice (Fig. 1). Habituation also had a main effect (*F*_1,96_ = 12.515, *P* = 0.001) with habituation leading to an increase in social activity. A main effect of sex was seen (*F*_1,96_ = 49.440, *P* < 0.001) as well as a sex × genotype interaction (*F*_1,96_ = 7.600, *P* = 0.007) but no other interaction terms were significant (genotype × habituation interaction *F*_1,96_ = 0.315, *P* = 0.576; sex × habituation interaction *F*_1,96_ = 0.334, *P* = 0.565; genotype × sex × habituation *F*_1,96_ = 0.554, *P* = 0.458). Pairwise comparisons showed the genotype × sex interaction was due to male WT mice interacting more than GluN1^hypo^ male mice [time spent socially interacting (mean ± SEM): WT 127.6 ± 11.2 s, GluN1^hypo^ 72.1 ± 10.5 s; *P* < 0.001, Fig. 1a] but no difference between genotypes was seen in female mice (WT 51.6 ± 4.6 s, GluN1^hypo^ 42.9 ± 4.7 s; *P* = 1.000, Fig. 1b). WT male mice interacted significantly more than WT female mice (*P* < 0.001) but only a trend for this sex difference was seen in GluN1^hypo^ mice (*P* = 0.066). WT male mice were seen to socially interact more than GluN1^hypo^ female mice (*P* < 0.001) but no difference was seen between WT female and GluN1^hypo^ male mice (*P* = 0.401).

Despite the lack of a genotype × habituation interaction, we investigated the effect of genotype within each habituation condition to address the *a priori* interest in whether habituation would rescue social behavior in the GluN1^hypo^ mice. In line with the overall effect of genotype and lack of an interaction, GluN1^hypo^ mice displayed reduced social behavior in both conditions relative to WT mice [no habituation, time spent socially interacting (mean ± SEM): WT 78.3 ± 10.4 s, GluN1^hypo^ 43.7 ± 8.7 s; *F*_1,50_ = 9.961, *P* = 0.003; following habituation, time spent socially interacting (mean ± SEM): WT 98.8 ± 11.6 s, GluN1^hypo^ 71.3 ± 7.0 s; *F*_1,46_ = 6.073, *P* = 0.018). Thus, GluN1^hypo^ mice displayed reduced levels of social interaction. We cannot rule out the possibility that different results might have been obtained if we had used an alternative paradigm in which WT and mutants had been individually paired with a partner from a neutral strain, although it is re-assuring that deficits in social behavior have been observed elsewhere with GluN1^hypo^ mice using such test parameters (Duncan *et al.* 2004; Halene *et al.* 2009; Mohn *et al.* 1999).

**Figure 2:**
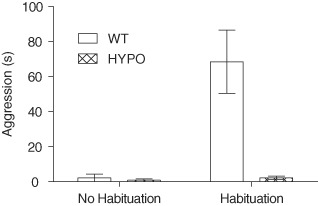
Time spent in aggressive encounters for male GluN1 ^hypo^(HYPO) and WT mice. In the social interaction task, male GluN1^hypo^ showed less aggressive behavior than WT mice. This was most apparent following habituation to the testing environment as a result of habituation leading to a large increase in aggressive behavior in the WT mice but not in the HYPO mice, with both groups showing very little aggressive behavior when not habituated to the testing context. Time spent in aggressive encounters is expressed as mean ± SEM.

Aggressive behavior was only seen in male mice and so female mice were excluded from the analysis. Aggression was also much less common than social interaction. Aggression was consistently very low in the GluN1^hypo^ mice both before and after habituation but increased markedly in WT mice following habituation (Fig. 2). Statistically, main effects of genotype (*F*_1,46_ = 14.628, *P* < 0.001) and of habituation (*F*_1,46_ = 14.703, *P* < 0.001) were seen as well as a genotype × habituation interaction (*F*_1,46_ = 13.476, *P* = 0.001). Pairwise comparisons showed habituation increased aggression in WT mice [time spent in aggressive encounters (mean ± SEM): no habituation 2.2 ± 2.2 s, following habituation 68.5 ± 18.0 s; *P* < 0.001] but not GluN1^hypo^ mice (no habituation 0.8 ± 0.8 s, following habituation 2.3 ± 0.9 s; *P* = 1.000). No genotype difference was seen before habituation (*P* = 1.000) but WT mice were significantly more aggressive than GluN1^hypo^ mice following habituation (*P* < 0.001). The slight increase in aggression in the GluN1^hypo^ mice following habituation was sufficient to make the level of aggressive encounters similar to non-habituated WT mice (*P* = 1.000). Habituated WT mice were significantly more aggressive than non-habituated GluN1^hypo^ mice (*P* < 0.001).

### Test of cognition

#### Discrete trial, spontaneous alternation (Enclosed T-maze)

**Figure 3:**
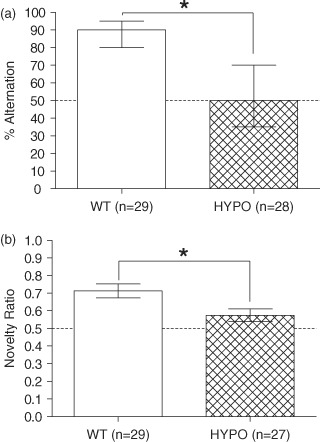
Performance of GluN1 ^hypo^(HYPO) and WT mice in discrete trial spontaneous alternation (a) and the spatial novelty Y-maze (b) tasks. In the spontaneous alternation task (a), WT mice show a high level of performance while GluN1^hypo^ mice were at chance levels. In the spatial novelty Y-maze (b), the GluN1^hypo^ mice once again showed deficits compared to the WT mice. Spontaneous alternation is expressed as median and IQR, spatial novelty Y-maze as mean ± SEM of the novelty preference ratio, defined as time spent in novel arm/(time spent in novel arm + time spent in other arm). *P < 0.05 WT vs. HYPO.

In spontaneous alternation, a test of short-term spatial memory, WT mice showed good performance levels, while GluN1^hypo^ mice performed at chance levels (group difference *U*_57_ = 63.500, *P* < 0.001; performance vs. chance WT *t* = 19.783, *P* < 0.001; GluN1^hypo^*t* = 0.987, *P* = 0.332, Fig. 3a).

#### Spatial novelty preference (Y-maze)

The spatial novelty preference Y-maze task, a further test of spatial, short-term memory, showed similar results to the discrete trial, spontaneous alternation task, with WT mice showing good performance levels and GluN1^hypo^ mice displaying a significant impairment but, in this case, slightly above chance levels of performance (group difference *F*_1,52_ = 7.067, *P* = 0.010; performance vs. chance WT *t* = 5.457, *P* < 0.001; GluN1^hypo^*t* = 2.086, *P* = 0.047, Fig. 3b). No effect of sex (*F*_1,52_ = 0.383, *P* = 0.539) or genotype × sex interaction (*F*_1,52_ = 1.415, *P* = 0.240) was seen.

#### Object recognition

**Figure 4:**
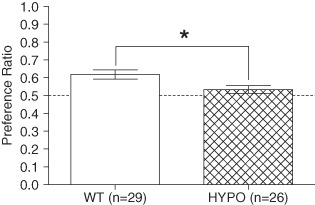
Performance of GluN1 ^hypo^(HYPO) and WT mice during the test phase of the novel object recognition task. GluN1^hypo^ mice were significantly impaired compared to WT mice. Performance is expressed as mean ± SEM of preference ratio, defined as time spent exploring novel object/(time spent exploring novel object + time spent exploring familiar object). *P < 0.05 WT vs. HYPO.

Object recognition was used as a test of non-spatial, short-term memory. WT mice showed a strong preference for the novel object during the test phase and this was not seen in the GluN1^hypo^ mice (group difference *F*_1,51_ = 6.660, *P* = 0.013; performance vs. chance WT *t* = 4.509, *P* < 0.001; GluN1^hypo^*t* = 1.561, *P* = 0.131, Fig. 4). An effect of sex was seen (*F*_1,51_ = 6.771, *P* = 0.012) with female mice (0.63 ± 0.02) performing better than male mice (0.54 ± 0.02), but this did not interact with genotype (*F*_1,51_ = 0.764, *P* = 0.386).

GluN1^hypo^ mice spent more time exploring the objects in total during the test phase [time spent exploring objects (mean ± SEM): WT 23.1 ± 2.02 s, GluN1^hypo^ 39.0 ± 4.05 s; *F*_1,51_ = 15.674, *P* < 0.001]. While no main effect of sex was seen (*F*_1,51_ = 2.108, *P* = 0.153) there was a genotype × sex interaction (*F*_1,51_ = 5.763, *P* = 0.020). Pairwise comparisons showed this to be due to GluN1^hypo^ female mice exploring more than WT female mice (WT 21.0 ± 3.3 s, GluN1^hypo^ 47.0 ± 7.2 s; *P* < 0.001), WT male mice (25.0 ± 2.4 s, *P* = 0.002) and showed a trend toward exploring more than GluN1^hypo^ male mice (31.5 ± 3.5 s, *P* = 0.064). No other significant differences were seen (WT male vs. WT female mice *P* = 1.000; WT male vs. GluN1^hypo^ male mice *P* = 1.000; WT female vs. GluN1^hypo^ male mice *P* = 0.471).

Importantly, exploration during the sample trial was not significantly different between the genotypes [time spent exploring objects (mean ± SEM): WT 56.7 ± 5.03 s, GluN1^hypo^ 69.7 ± 6.80 s; *F*_1,51_ = 2.581, *P* = 0.114] and was not affected by sex (effect of sex *F*_1,51_ = 0.645, *P* = 0.426; genotype × sex interaction *F*_1,51_ = 1.421, *P* = 0.239).

#### Spatial reference memory (elevated Y-maze)

**Figure 5:**
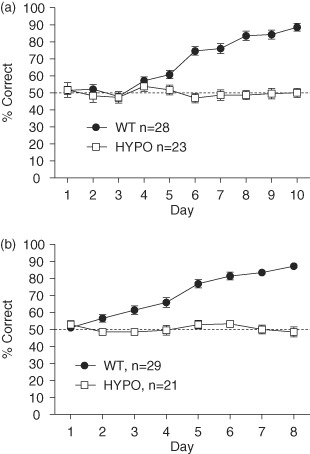
Performance of GluN1 ^hypo^(HYPO) and WT mice in the spatial reference Y-maze (a, mean ± SEM) and visual discrimination (b, mean ± SEM) tasks. GluN1^hypo^ mice failed to acquire either the spatial or non-spatial associative long-term memory task.

GluN1^hypo^ mice displayed a striking spatial reference memory impairment on the appetitively motivated, elevated Y-maze task. Whereas WT mice gradually learned to choose the rewarded goal arm, the GluN1^hypo^ mice remained at chance levels (Fig. 5a). Statistical analysis showed a main effect of day (*F*_9,423_ = 17.789, *P* < 0.001), genotype (*F*_1,47_ = 59.805, *P* < 0.001) and a day × genotype interaction (*F*_9,423_ = 19.617, *P* < 0.001). Simple main effects analysis showed that a main effect of day was only present in WT mice (*F*_9,39_ = 17.914, *P* < 0.001), and not in GluN1^hypo^ mice (*F*_9,39_ = 0.885, *P* = 0.547). This shows an incremental improvement in performance in the WT mice across training, but provides no evidence for learning in the GluN1^hypo^ mice. Performance in the GluN1^hypo^ mice was also never statistically above chance on any day of the task (*P* = 1.000 − 0.205), whereas WT performance was above chance from day 4 (*P* = 0.004) and on all subsequent days (all *P* < 0.001). Sex did not affect acquisition of the spatial reference memory Y-maze test (effect of sex *F*_1,47_ = 3.277, *P* = 0.077; genotype × sex interaction *F*_1,47_ = 0.150, *P* = 0.700; day × sex interaction *F*_9,423_ = 1.673, *P* = 0.093; genotype × day × sex interaction *F*_9,423_ = 0.515, *P* = 0.863).

#### Visual discrimination learning

GluN1^hypo^ mice also failed to acquire a simple, non-spatial visual discrimination task. Whereas WT mice improved progressively with training, the GluN1^hypo^ mice again remained at chance levels of performance (Fig. 5b). Main effects of day (*F*_7,322_ = 23.386, *P* < 0.001) and genotype (*F*_1,46_ = 98.630, *P* < 0.001), as well as a day × genotype interaction (*F*_7,322_ = 22.328, *P* < 0.001), were seen. Simple main effects showed acquisition of the task in WT mice (*F*_7,40_ = 51.353, *P* < 0.001) but not in the GluN1^hypo^ mice (*F*_7,40_ = 0.918, *P* = 0.503). Performance in the GluN1^hypo^ mice was also never statistically above chance on any day of the task (*P* = 1.000 − 0.069), whereas WT performance was above chance from day 2 (*P* = 0.006) and on all subsequent days (all *P* < 0.001). Sex had no effect on acquisition of this task (effect of sex *F*_1,46_ = 0.294, *P* = 0.590; genotype × sex interaction *F*_1,46_ = 0.007, *P* = 0.932; day × sex interaction *F*_7,322_ = 1.334, *P* = 0.233; genotype × day × sex interaction *F*_7,322_ = 1.026, *P* = 0.412).

## Discussion

NMDA hypofunction has been implicated in the pathogenesis of schizophrenia. Previous studies have shown that GluN1^hypo^ mice, constitutively expressing 5–10% of WT levels of functional NMDA receptors, exhibit behaviors analogous to some of the symptoms of this psychiatric disease. This study shows that these GluN1^hypo^ mice in fact display greatly altered behavior, including profound performance deficits in several cognitive tasks. Performance was impaired on both spatial and non-spatial tests of both short- and long-term memory. This suggests that these mice have more global alterations in behavior than previously realized, and may not selectively phenocopy any individual human psychiatric disease.

Although this study is by no means exhaustive and cannot encompass every learning and memory domain, profound and enduring deficits were seen in all cognitive tasks performed. Indeed, there was little evidence of learning in the GluN1^hypo^ mice on any of the tasks, with performance levels rarely above chance. Thus, GluN1^hypo^ mice displayed robust impairments in both short- and long-term memory, on both spatial and non-spatial tasks. Such wide-ranging behavioral deficits have also been noted in mice lacking the GluN2B NMDA receptor subunit from principal cells across the forebrain (von Engelhardt *et al.* 2008). In such cases, it is not possible to dissociate a mnemonic phenotype from a more general performance deficit. In the absence of a control task on which GluN1^hypo^ mice display normal performance we are not able to rule out the possibility that observed deficits on the learning and memory tasks are due to altered sensorimotor or motivational processes.

For example, the deficits found in cognitive tests could be due to altered motivation in the GluN1^hypo^ mice. However, as pointed out above, it is impossible to attribute any of the behavioral phenotypes in these mice to any specific psychological or behavioral process. The increased sucrose preference of these mice might suggest some alteration in motivation, but a decrease in this measure is what is normally considered to represent anhedonia, which might be considered as a potential explanation for the global impairment on appetitively motivated tasks. Furthermore, the GluN1^hypo^ mice were actually found to consume more food than WT mice. These measures do not obviously suggest that the GluN1^hypo^ mice would be less motivated in appetitively reinforced tasks. Importantly, robust deficits were also found in cognitive tasks that rely on spontaneous behavior, such as object recognition and spontaneous alternation. This all suggests that, while motivational changes could conceivably exist, these are not sufficient to explain all of the deficits seen in the GluN1^hypo^ mice.

A further possible confound for all tests performed is effect of test order and the number of tests each animal was exposed to. While we cannot rule out a possible contribution, no clear carry over effect was seen in the tests. For example, the GluN1^hypo^ mice did not appear to get progressively more anxious in subsequent tests. It therefore seems unlikely that this can fully explain the results obtained.

The results of the cognitive tests are very difficult, if not impossible, to interpret, other than to conclude that NMDA receptors make an important contribution, at one level or another, to a great many behaviors, which is hardly surprising given their widespread distribution throughout the brain. This of course highlights the importance of region-specific GluN1 deletions, using tissue specific promoters (Nakazawa *et al.* 2002, 2003; Tsien *et al.* 1996a, b). For example, very specific cognitive deficits have been seen following deletion of the GluN1 subunit selectively from the granule cells of the dentate gyrus (Niewoehner *et al.* 2007). GluN1^ΔDG^ mice display impairments in spatial working memory, while at the same time, showing preserved spatial reference memory performance, indicating a very specific role for NMDA receptors, in a particular brain region, in a particular aspect of cognition.

The results of the anxiety tests are also difficult to interpret, with no clear picture emerging from the various tests performed in this study. The excessive hyperactivity observed in the GluN1^hypo^ mice is likely to be a major confound on the anxiety test battery, although it is difficult to predict from one test to the next whether hyperactivity will manifest as behavioral differences which look like an increase or a decrease in anxiety (see also Fitzgerald *et al.* 2010). Indeed, the data from the anxiety tests likely reflect a complex set of interactions between various behavioral processes, that differ from one test to the next, underscoring the complexity of these ostensibly simple mouse behavioral tasks that are commonly employed, but not always carefully interpreted (Holmes 2001). As with the cognitive tests, the use of region-specific GluN1 deletions are likely to be more informative (Barkus *et al.* 2010).

The severity and wide-ranging nature of the behavioral impairment in GluN1^hypo^ mice is difficult to reconcile with its putative role as an animal model of schizophrenia. GluN1^hypo^ mice show profound deficits in tests of short- and long-term memory, on both spatial and non-spatial learning tasks, as well as disruptions to naturalistic behaviors. When viewed against this background of far-reaching behavioral alterations, the specificity of the deficits in PPI and social interaction reported previously may need to be reconsidered. Indeed, the present data from GluN1^hypo^ mice are indicative of a global impairment rather than any specific disease.

That is not to dispute that NMDA receptor hypofunction is a key feature of schizophrenia (Javitt 2007), and a targeted reduction of GluN1, limited to particular brain regions, particular cell groups, or at a particular stage of development may result in a more selective behavioral model. For example, Belforte and colleagues recently generated a mouse line with the post-developmental removal of approximately 50% of the NMDA receptors selectively from interneurones within the cortex and hippocampus (Belforte *et al.* 2010). When this was performed before adolescence, a host of schizophrenia-like behaviors were seen, but not when the reduction in NMDA receptors was only evident later on into adulthood. The changes in behavior seen included deficits in PPI, social interaction, as well as spatial working memory as assessed by spontaneous alternation. This elegantly shows not only a role for NMDA receptors in schizophrenia-like phenotypes but also the importance of temporal specificity in the manipulations used. The model used by Belforte and colleagues may therefore provide an NMDA-driven model in which phenotypes relevant to schizophrenia are seen in isolation from global cognitive deficits, although it is important to point out that this is also yet to be fully tested. In contrast, the use of the GluN1^hypo^ mice as a model of schizophrenia (Duncan *et al.* 2004, 2006a,b; Fradley *et al.* 2005; Halene *et al.* 2009; Mohn *et al.* 1999) may need to be interpreted with caution in light of the gross alterations in behavior seen in this study.
